# Reproductive disorders among cosmetologists and hairdressers: a meta-analysis

**DOI:** 10.1007/s00420-016-1112-z

**Published:** 2016-01-28

**Authors:** Dohyung Kim, Mo-Yeol Kang, Sungyeul Choi, Jaechan Park, Hye-Ji Lee, Eun-A. Kim

**Affiliations:** Occupational Safety and Health Research Institute, 400, Jongga-ro, Jung-gu, Ulsan, 681-230 South Korea; Department of Preventive Medicine, College of Medicine, Seoul National University, Seoul, South Korea

**Keywords:** Reproductive disorder, Hairdresser, Cosmetologist, Meta-analysis

## Abstract

**Purpose:**

Occupational risks for reproductive disorders among hairdressers and cosmetologists have been examined in numerous epidemiological studies, although the results of those studies have been inconsistent. Therefore, we conducted a meta-analysis of published studies to evaluate the risks of reproductive disorders among cosmetologists and hairdressers.

**Methods:**

We searched the MEDLINE, EMBASE, and Cochrane Library databases, as well as the reference lists of relevant publications, to identify studies for our analysis. After careful consideration, 19 eligible studies were included in the meta-analysis. We also performed systematic evaluations of publication bias, heterogeneity, and publication quality.

**Results:**

Study-specific odds ratios (ORs) were weighted using the inverse of their variance to calculate fixed- and random-effect pooled estimates. The meta-analysis revealed a significantly increased risk of infertility (OR 1.15, 95 % CI 1.03–1.28), fetal death (OR 1.14, 95 % CI 1.04–1.24), and preterm delivery (OR 1.04, 95 % CI 1.00–1.07) among hairdressers and cosmetologists.

**Conclusion:**

These findings indicate that hairdressers and cosmetologists have a higher risk of reproductive disorders, compared to the general population.

## Introduction

Cosmetologists are generally defined as individuals who work in retail- or home-based salons and provide a wide range of beauty services, including hair shampooing and styling, manicures, pedicures, and scalp and facial treatments. Hairdressing and cosmetology are common occupations, and several million individuals are employed as hairdressers and cosmetologists (HC) worldwide (European Agency for Safety and Health at Work 2014). Workers in the hairdressing and cosmetology professions are predominantly women, and many of these women are of childbearing age (Halliday-Bell et al. 2009) and begin working before considering family planning (Baste et al. 2008). Therefore, this situation raises concerns that these women of reproductive age could be susceptible to the effects of exposure to potential reproductive toxins.

Hairdressers can be exposed to a variety of chemicals on a daily basis, due to their use of hair products, shampoos, permanent wave solutions, hair dyes, and hair sprays (Labrèche et al. 2003; Jung et al. 2014). Reproductive toxic effects have been reported for some of these agents (Rylander et al. 2002; Rylander and Källén 2005; Pak et al. 2013; Quach et al. 2014), including selenium, some dye formulations, and lead acetate (in animals), in addition to organic solvents, nitrosamines, formaldehyde, dibutyl phthalate, ethylene glycol ethers, and hexachlorophene (in human patients).

In addition to the related chemical agent exposure, work as a HC consists of prolonged periods of bending and standing, as well as work-related stress, which may have unfavorable effects on reproduction (Strine et al. 2005; Palmer et al. 2013). Furthermore, successful hair salons require a “customer-focused” workplace environment, although the customers’ needs must be balanced with the understanding that healthy employees provide the best service. Moreover, HC’s working hours may vary according to the customers’ demands, and a high level of concentration and punctuality is needed to achieve customer satisfaction. The work is also frequently performed at a high pace and under considerable time pressures and other stressful conditions (Ronda et al. 2010). Finally, HC are self-employed with few employees, which can create an environment with limited support for managing workplace health and safety. Taken together, these ergonomic, chemical, and psychosocial factors have generated concern regarding adverse pregnancy outcomes among HC (Kersemaekers et al. 1997).

Several studies of HC have suggested that their work might adversely affect their reproductive health (Herdt-Losavio et al. 2009; Ronda et al. 2010; Jørgensen et al. 2013; Quach et al. 2014), although various studies have reported conflicting findings. For example, several studies have reported that HC have an increased risks of infertility (Baste et al. 2008), a time to pregnancy of >12 months (Kersemaekers et al. 1997), spontaneous abortion (Ronda et al. 2010), low birth weight (Halliday-Bell et al. 2009; Herdt-Losavio et al. 2009), and preterm delivery (Halliday-Bell et al. 2009), compared to women in other occupations or in the general population. However, other studies have found little or no evidence of an increased reproductive health risk among female hairdressers (Hougaard et al. 2006; Gallicchio et al. 2011).

One review article has stated that an increased risk of fertility disorders and pregnancy complications among HC cannot be excluded (Peters et al. 2010). However, although that study’s authors summarized the available evidence regarding fertility disorders and pregnancy complications among HC, they did not perform a comprehensive meta-analysis. Nevertheless, despite the lack of decisive supportive evidence regarding reproductive toxicity among HC, concerns persist regarding their occupation-related safety, especially among pregnant HC. Therefore, we conducted this meta-analysis to determine whether HC have an increased risk of reproductive disorders, such as small for gestational age (SGA), low birth weight (LBW), infertility, preterm birth, and fetal death.

## Methods

### Search strategy

We searched the MEDLINE, EMBASE, and Cochrane Library databases for studies that reported reproductive disorders among HC between January 1970 and January 2015. The search terms that we used were as follows: “female” or “woman”; “hairdresser” or “hairstylist” or “cosmetologist”; and “spontaneous abortion” or “fetal/early loss” or “fetal death” or “preterm/perinatal death” or “stillbirth” or “small for gestational age” or “fertility” or “infertility” or “subfertility” or “time to pregnancy” or “preterm delivery” or “low birth weight” or “pregnant/reproductive disorder.” We also manually searched the reference lists of the relevant articles that were obtained from our search. Studies were considered eligible and included in the analysis if they met all of the following criteria: (1) case–control, cohort, or cross-sectional design; (2) reported effect estimates, such as odd ratios (OR) and relative risk (RR) with 95 % confidence interval (CI), or outcome values that allowed for effect estimate calculations in a 2 × 2 cell table; and (3) discussed whether the mother’s work as a HC was associated with their adverse pregnancy outcomes. The exclusion criteria were as follows: (1) reported irrelevant outcomes; (2) absence of effect estimates or if we could not calculate the risk; (3) letter, comment, or review article; (4) identical study population; (5) not written in English; and (6) not human subjects. If a study population was duplicated in more than one article, we included only the latest publication after a review of the full text. However, studies were included if different outcome variables were reported, despite the use of identical study populations. Two authors (DK and MK) screened all of the abstracts, reviewed the full texts, and determined eligibility according to the inclusion criteria; discrepancies regarding a study’s inclusion were resolved via discussion and consensus.

### Bias and confounding variable evaluation

All included studies adjusted for maternal age as a confounding variable in the final model, but only eight studies adjusted for parity/gravidity, whereas 15 adjusted for maternal smoking. The adjusted confounding variables in the included studies are listed in Table 1. To control potential selection bias, most studies compared the general characteristics of the study group with a reference group, but four studies did not (Li et al. 2010; Rylander and Källén 2005; McDonald et al. 1987, 1988). To prevent recall bias, some studies compared the answers of the subject’s questionnaire with hospital records or birth certificates (Herdt-Losavio et al. 2011; Ronda et al. 2010). The potential for recall bias is also indicated in Table 1.

**Table 1:** Characteristics of the included studies

Reference, year, country	Study design	Follow-up/study period	Study population	OR or RR (95 % CI)					Potential for recall bias	Adjusted confounding variables
				SGA	LBW	Infertility	Fetal death	Preterm delivery		
Registry-based studies										
Quach et al. (2014), USA	Cohort	1996–2009	56,373 cosmetologists 24,832 manicurists 53,056 other working group^a^	0.96^b^ (0.91–1.00)	0.98 (0.92–1.04)	–	–	1.01 (0.97–1.06)	Low	Age, parity, race, birth order, education, month prenatal care began
				0.98^b^ (0.91–1.05)	1.05 (0.96–1.16)	–	–	1.07 (0.99–1.15)		
Li et al. (2010), Sweden	Cohort	1990–2004	210 SGA of hairdresser 29,603 SGA of all mothers^a^	1.21 (0.97–1.51)	–	–	–	–	Low	Age, period of birth, family income, region of residence, marital status, smoking habits
Herdt-Losavio et al. (2009), USA	Cohort	1997–2003	15,003 cosmetologists 4246 realtors ^a^	1.10 (0.93–1.30)	1.38 (1.09–1.74)	–	–	0.97 (0.83–1.12)	Low	Age, parity ethnicity, education, race, employment, BMI, smoking, participation in any aid program, prenatal care, alcohol use, diabetes
Halliday-Bell et al. (2009), Finland	Cohort	1990–2004	10,622 singletons of hairdressers 2490 singletons of cosmetologists 18,594 singletons of teachers^a^	1.65^c^ (1.38–2.07)	1.44 (1.23–1.69)	–	1.62 (1.01–2.60)	1.21 (1.07–1.38)	Low	Age, parity, marital status, smoking
				1.53^c^ (1.10–2.12)	1.20 (0.92–1.58)	–	1.36 (0.62–2.98)	0.90 (0.72–1.13)		
Axmon and Rylander (2009), Sweden	Cohort	1982–2005	3137 hairdressers 3952 sisters^a^	0.80 (0.49–1.31)	0.83 (0.56–1.21)	–	–	–	Low	Age, parity, smoking, height, infant sex
Hougaard et al. (2006), Denmark	Cohort	1998–2002	4113 hairdressers 33,775 shop assistants^a^	–	–	1.01 (0.77–1.29)	–	–	Low	Country, social group
Zhu et al. (2006), Denmark	Cohort	1997–2003	550 hairdressers 3216 shop assistants^a^	1.0 (0.7–1.3)	–	–	0.7 (0.3–1.8)	1.0 (0.7–1.6)	Low	Age, gravidity, history of spontaneous abortion, BMI, smoking, alcohol
Rylander and Källén (2005), Sweden	Cohort	1983–2001	12,046 infants of hairdressers 1280,791 deliveries from all other working mothers^a^	1.19 (1.07–1.33)	1.10 (0.99–1.21)	–	–	1.05 (0.96–1.14)	Low	Age, parity, year of birth, smoking
McDonald et al. (1988), Canada	Case–control	1982–1984	22,613 pregnancies among all workers ^a^ 354 pregnancies of hairdressers	–	–	–	1.02 (0.84–1.23)	–	Low	Age, gravidity, education, spouse’s education, smoking, alcohol, ethnicity, height, previous spontaneous abortion, previous premature birth
McDonald et al. (1987), Canada	Case–control	1982–1984	688 pregnancies of hairdressers 46,628 pregnancies among all workers^a^	–	1.20 (0.90–1.61)	–	–	–	Low	Age, gravidity, education, spouse’s education, smoking, alcohol, ethnicity, height, previous spontaneous abortion, previous premature birth
Questionnaire-based studies										
Herdt-Losavio et al. (2011), USA	Case–control	1997–2003	125 LBW infants of hairdressers 159 normal birth weight infants of hairdressers^ae^	–	1.43 (0.82–2.49)	–	–	–	Low	Age, year of birth, race, ethnicity, use of government assistance programs, smoking, alcohol, standing for work
Ronda et al. (2010), Spain	Cross-sectional	2006	94 hairdressers 138 shop assistants and office workers^a^	–	0.2 (0.3–2.0)	–	1.6 (0.9–2.7)	1.0 (0.4–2.9)	Low	Age
Ronda et al. (2009), Spain	Cross-sectional	2006	310 hairdressers 310 shop assistants and office workers^a^	–	–	2.17 (0.91–5.17)	–	–	High	Age, smoking
Peretz et al. (2009), USA	Cross-sectional	2005–2008	448 cosmetologists 508 non-cosmetology workers^a^	–	–	0.82 (0.57–1.17)	–	–	High	Age, race, education, BMI, marital status, smoking, alcohol
Gallicchio et al. (2009), USA	Cross-sectional	2005–2008	350 cosmetologists 397 other occupations^a^	–	0.61 (0.29–1.27)	–	1.03 (0.74–1.43)	0.64 (0.37–1.13)	High	Age, race, education, smoking, alcohol
Baste et al. (2008), Norway	Cross-sectional	1997–1999	221 hairdressers 10,291 other occupations^a^	–	–	1.30 (1.08–1.55)	1.31 (1.07–1.60)	–	High	Age, education, smoking
Axmon et al. (2006), Sweden	Cohort	Until 2000	2626 hairdressers 2860 general population^a^	–	–	1.10 (0.93–1.39)	1.12 (0.88–1.42)	–	High	Age, year of birth, performance of heavy lifts, use of oral contraceptives, menstrual cycle length, partner’s smoking habit, workplace smoking
Kersemaekers et al. (1997), Netherlands	Cohort	1986–1993	9000 hairdressers 9000 clothing salesclerks^a^	–	1.5^d^ (0.7–3.1)	1.5 (0.8–2.8)	1.6 (1.0–2.4)	0.7 (0.1–2.2)	High	Age, gravidity, educational level
				–	1.2^d^ (0.8–1.9)	1.2 (0.8–1.6)	0.9 (0.7–1.1)	1.0 (0.8–1.4)		
John et al. (1994), USA	Cross-sectional	1983–1988	96 spontaneous abortion of cosmetologists 547 single live births of cosmetologists^a,f^	–	–	–	1.4 (0.8–2.3)	–	High	Age, smoking, previous pregnancy loss

*OR* odds ratio, *RR* relative risk, *CI* confidence interval, *SGA* small for gestational age, *LBW* low birth weight, *BMI* body mass index

^a^Reference population

^b^Upper line for cosmetologists and lower line for manicurists

^c^Upper line for hairdressers and lower line for cosmetologists

^d^Upper line for 1986–1998 and lower line for 1991–1993

^e^Risk factor is hours worked per week as a hairdresser in the 3 months before and during pregnancy, >30 h

^f^Risk factor is hours worked per week as a cosmetologist during the first trimester of pregnancy, ≥35 h

### Quality assessment

The Newcastle–Ottawa scale (NOS) was used to evaluate the quality of the included studies (Wells et al. 2014). For each study, we rated 9 items using a score of 0 or 1, and the total score was calculated to determine the study’s quality (possible range 0–9).

For case–control and cross-sectional studies, this scoring system evaluated 4 items for the selection of cases and controls, 2 items for the comparability of cases and controls, and 3 items to ascertain exposure. For example, item 1 was rated for adequate case definition with independent validation or reference to primary record source, such as medical/hospital records, but not for simple record linkage to a database or self-report. Item 5 was rated for controlling the most important potentially confounding variable (i.e., previous reproductive history); therefore, if a study only analyzed the first pregnancy or used specific statistical methods that dealt with correlated observations, the study could be rated. Item 6 was rated for adjusting the regression model for the second most important confounding variables, such as maternal age and parity/gravidity. Item 7 was rated for quantitative exposure assessment, which was described as working time per week during pregnancy.

For cohort studies, this scoring system evaluated 4 items for the selection of the cohorts, 2 items for comparability, and 3 items for the assessment of outcome. For example, item 3 for ascertainment of exposure was rated only if the study provided quantitative exposure assessment, such as the working time as a hairdresser or cosmetologist during pregnancy. Items 5 and 6 for comparability were the same as those in case–control studies. Item 7 for the assessment of outcome was rated when independent or blind assessment was reported in the paper, or when the outcome was confirmed by reference to medical/hospital records or record linkage to a database. More detailed information regarding how the ratings were applied has been previously reported (Wells et al. 2014).

### Data extraction

The data that we extracted included the data source, study design, authors, publication year, country of origin, data collection period, definition of case–controls or cohorts, types of adverse pregnancy outcome(s), and adjusted effect estimates. Several studies reported effect estimates as observed to expected (*O*/*E*) ratios, and we manually calculated the relative risks and confidence intervals using 2 × 2 cell tables (Morris and Gardner 1988). All data were extracted exclusively from the published articles, and we did not contact the authors to obtain any additional information. Any data discrepancies were resolved via consensus among the authors.

### Statistical analysis

Our meta-analysis was conducted by grouping studies according to 5 outcomes: SGA, LBW, infertility, preterm delivery, and fetal death. In our analysis, fetal death was defined as intrauterine fetal death, including spontaneous abortion, preterm/perinatal death, or stillbirth. If a study reported the outcomes for two or more different groups that were compared to a common reference group, we estimated the common risks for the different groups, which were calculated using inverse-variance-weighted average. To calculate the overall OR, we attempted to use the outcome results in the final models and the 95 % CI that was described in each study. We also assessed inter-study heterogeneity using Cochran’s *Q* test and Higgins *I*^2^ statistic. If the *p* value of the Cochran’s *Q* test was <0.10, or if the Higgins *I*^2^ statistic was >50 %, we concluded that substantial inter-study heterogeneity was present and applied the random-effect (RE) model to calculate the overall OR. If substantial heterogeneity was not present, we used the fixed-effect (FE) model. Supplementary sub-analyses were conducted, in addition to an analysis of registry-based and questionnaire-based studies. First, we performed a separate analysis of hairdressers and cosmetologists, as their exposures may not be comparable. Similarly, sub-analyses according to the studies’ quality and potential for recall bias were also conducted. Publication bias was tested using Begg’s rank correlation test, and Egger’s regression test for funnel plot asymmetry. If the *p* value for either test was <0.05, we concluded that publication bias existed. All statistical analyses were performed using R software (version 3.1.2) and the “metafor” package (Viechtbauer 2010).

## Results

**Fig. 1:**
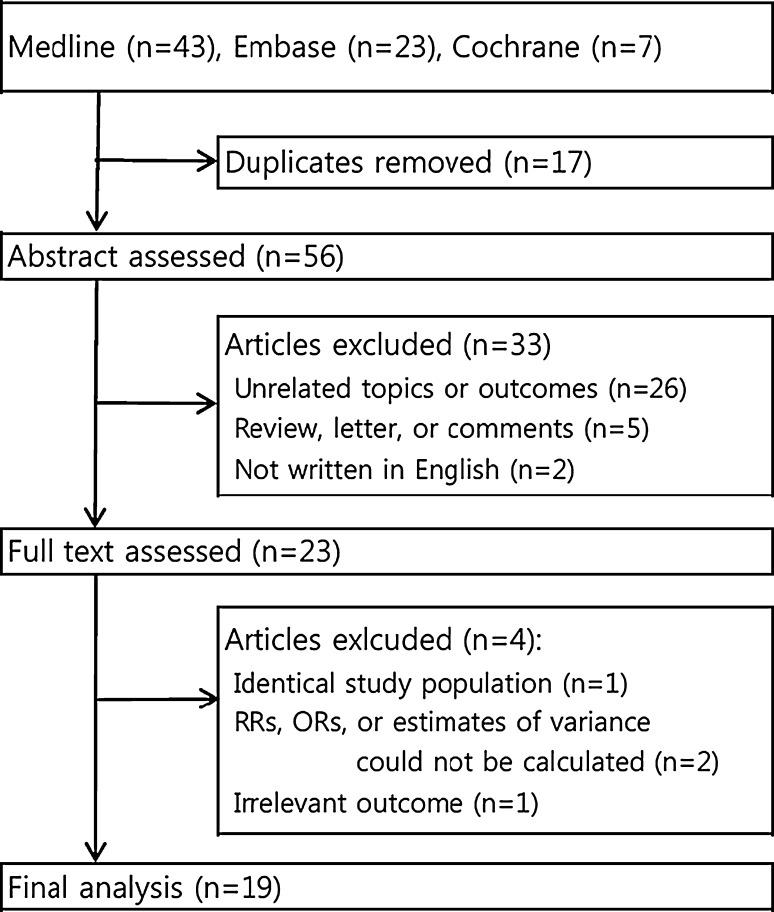
Flow diagram for identifying eligible studies. *RR* relative risk, *OR* odds ratio

Our search retrieved 73 studies from the three databases, as shown in Fig. 1. After screening the titles and abstracts, 50 studies were excluded due to duplication (*n* = 17); irrelevant outcomes (*n* = 26); review, letter, or comment articles (*n* = 5); and non-English language (*n* = 2). After the full-text review, we subsequently excluded 1 study that shared a common study population, 2 studies for which we could not calculate the effect estimates, and 1 study that reported an irrelevant outcome. Therefore, 19 eligible studies were included in the meta-analysis (10 cohort, 6 cross-sectional, and 3 case–control), and their characteristics and quality assessment scores are listed in Tables 1 and 2. We also classified the included studies as registry-based studies, which used national-/state-wide registries to identify reproductive outcomes by linking occupational and medical/birth records, or as questionnaire-based studies, which used in person/mail/telephone questionnaires/interviews to identify reproductive outcomes.

**Table 2:** Quality assessment according to the Newcastle–Ottawa scale

Eligible studies	Selection				Comparability		Ascertainment of exposure/outcome			Total score
	Item 1	Item 2	Item 3	Item 4	Item 5	Item 6	Item 7	Item 8	Item 9	
Registry-based										
Quach et al. (2014), USA	*	*		*		*	*	*	*	7
Li et al. (2010), Sweden	*			*	*	*	*	*	*	7
Herdt-Losavio et al. (2009), USA	*	*		*		*	*	*	*	7
Halliday-Bell et al. (2009), Finland	*	*		*		*	*	*	*	7
Axmon and Rylander (2009), Sweden	*	*		*		*	*	*	*	7
Hougaard et al. (2006), Denmark	*	*		*			*	*	*	6
Zhu et al. (2006), Denmark	*	*	*	*	*	*	*	*	*	9
Rylander and Källén (2005), Sweden	*		*			*	*	*	*	6
McDonald et al. (1988), Canada	*	*				*	*	*		5
McDonald et al. (1987), Canada	*	*				*	*	*		5
Questionnaire-based										
Herdt-Losavio et al. (2011), USA	*	*	*			*	*	*	*	7
Ronda et al. (2010), Spain	*	*	*	*	*	*	*	*	*	9
Ronda et al. (2009), Spain		*	*	*		*	*	*	*	7
Peretz et al. (2009), USA		*	*				*	*	*	5
Gallicchio et al. (2009), USA		*	*	*	*	*		*	*	7
Baste et al. (2008), Norway		*	*	*				*	*	5
Axmon et al. (2006), Sweden	*	*		*	*	*		*	*	7
Kersemaekers et al. (1997), Netherlands	*	*	*	*	*	*		*	*	8
John et al. (1994), USA		*	*	*		*	*	*	*	7

*For case–control or cross-sectional studies* Item 1: adequate case definition, Item 2: representativeness of the cases, Item 3: selection of controls, Item 4: definition of controls, Item 5: control for the most important factor, Item 6: control for any additional factor, Item 7: ascertainment of exposure, Item 8: same methods of ascertainment for cases and controls, Item 9: non-response rate. *For cohort studies*: Item 1: representativeness of the exposed cohort, Item 2: selection of the non-exposed cohort, Item 3: ascertainment of exposure, Item 4: outcome was not present at start of the study, Item 5: control for the most important factor, Item 6: control for any additional factor, Item 7: assessment of outcome, Item 8: follow-up long enough for outcome to occur, Item 9: adequacy of follow-up of cohorts

* Positive score for the indicated item

### Small for gestational age

**Fig. 2:**
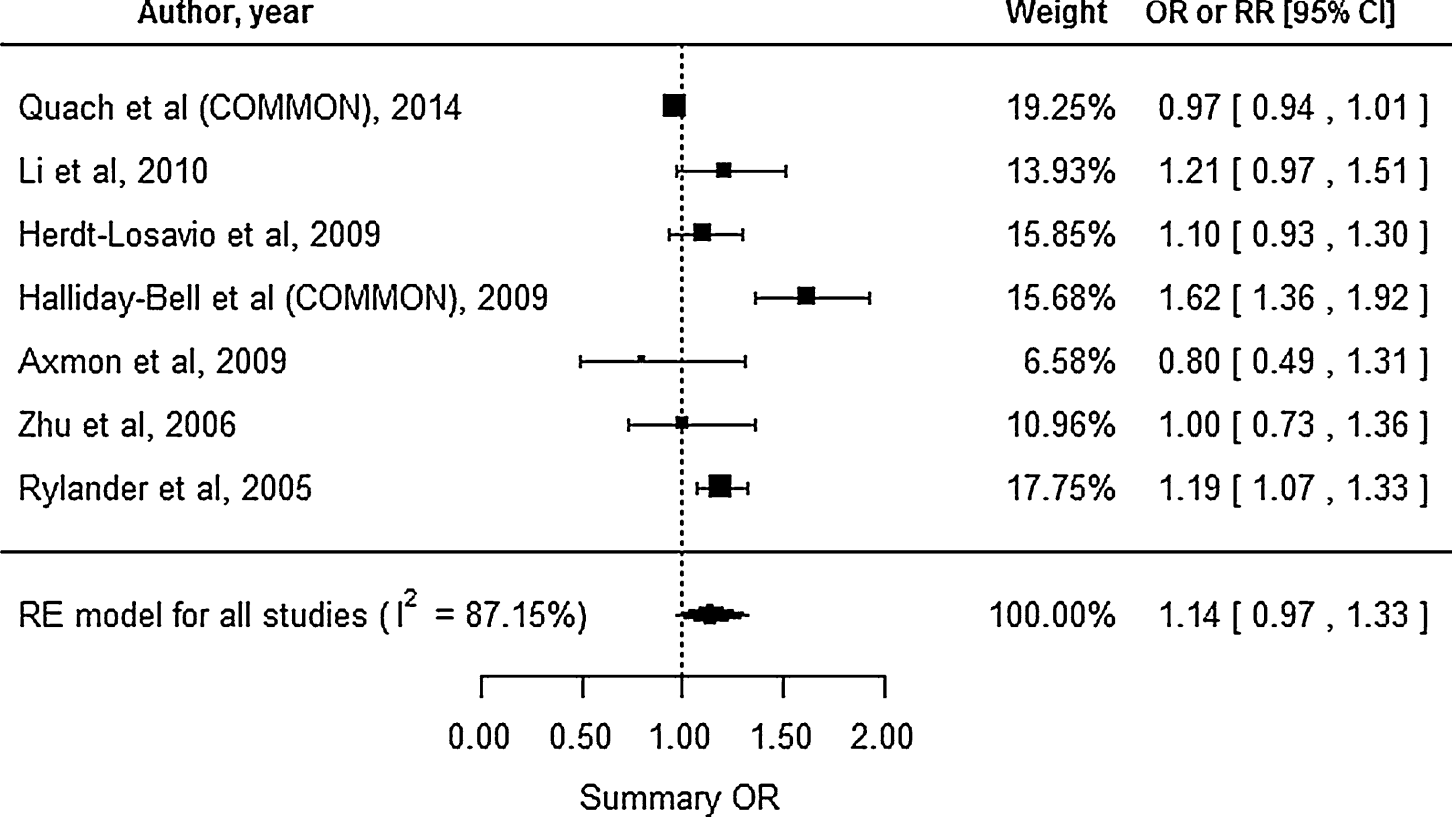
Forest plot for our meta-analysis of small for gestational age. *CI* confidence interval, *RE* random-effect, *OR* odds ratio, *RR* relative risk, *COMMON* common risk estimated

A total of 7 studies reported the SGA outcome, and all of these studies were registry-based (Fig. 2). The study by Quach et al. (2014) reported the outcomes for two different groups: cosmetologists and manicurists; therefore, we estimated the common risk for those groups. Similarly, we estimated the common risk for the study by Halliday-Bell et al. (2009), because the study reported outcomes for cosmetologists and hairdressers. Substantial heterogeneity was observed (*I*^2^ = 87.15 %, Cochran’s *Qp* value <0.0001), and we used the RE model to calculate the effect estimates. This analysis revealed an insignificantly increased summary OR of 1.14 (95 % CI 0.97–1.33) for SGA among HC. Begg’s test (*p* = 0.7726) and Egger’s test (*p* = 0.5611) did not reveal significant publication bias.

### Low birth weight

**Fig. 3:**
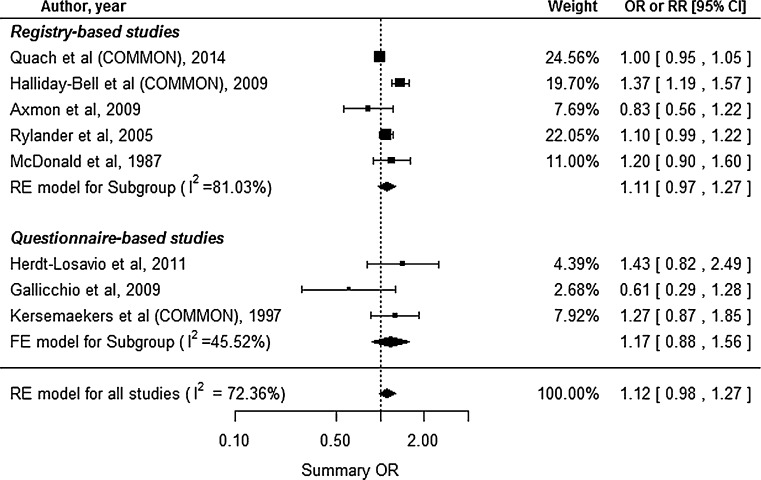
Forest plot for our meta-analysis of low birth weight. *CI* confidence interval, *RE* random-effect, *FE* fixed-effect, *OR* odds ratio, *RR* relative risk, *COMMON* common risk estimated

Eight studies reported the LBW outcome, including 5 registry-based and 3 questionnaire-based studies (Fig. 3). The studies by Quach et al. (2014), Halliday-Bell et al. (2009), and Kersemaekers et al. (1997) reported the outcomes for different groups or study periods: cosmetologists and manicurists, cosmetologists and hairdressers, and 1988–1991 and 1991–1993. Thus, we estimated common risks for each of the studies. Substantial heterogeneity was observed (*I*^2^ = 72.36 %, Cochran’s *Qp* = 0.0007), and we used the RE model for this analysis. The meta-analysis for LBW revealed a 12 % increase in the risk among HC, which was not statistically significant (95 % CI 0.98–1.27). However, the study by Ronda et al. (2010) was excluded from the analysis, because the outcome (OR) was incorrectly outside the confidence interval. We subsequently used Morris and Gardner’s (1988) methods to manually include Ronda et al.’s study in the RE model and found that the increased risk was similar to the original result (summary OR 1.11, 95 % CI 0.97–1.26). Subgroup analysis of the registry- and questionnaire-based studies also revealed increased risks of LBW among HC (summary ORs 1.11 and 1.17, respectively), which were not statistically significant (95 % CIs 0.97–1.27 and 0.88–1.56, respectively). Begg’s test (*p* = 1.0) and Egger’s test (*p* = 0.6439) did not reveal significant publication bias.

### Infertility

**Fig. 4:**
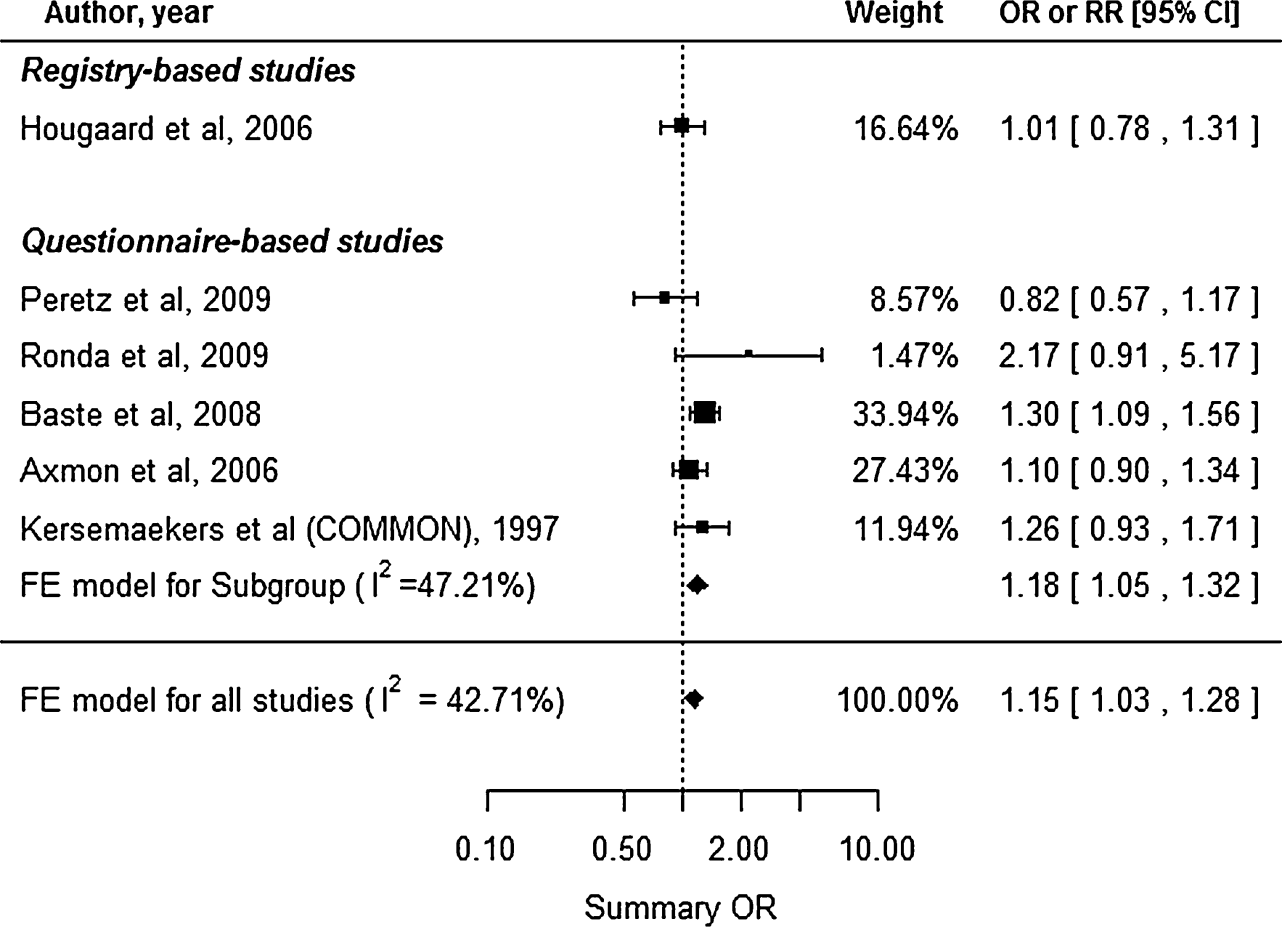
Forest plot for our meta-analysis of infertility. *CI* confidence interval, *FE* fixed-effect, *OR* odds ratio, *RR* relative risk, *COMMON* common risk estimated

Six studies reported the infertility outcome, including 1 registry-based and 5 questionnaire-based studies (Fig. 4). The study by Kersemaekers et al. (1997) reported the outcomes for two different study periods, and we estimated the common risk for that study. No substantial heterogeneity was observed (*I*^2^ = 42.71 %, Cochran’s *Qp* = 0.1204), and we used the FE model. Because the study by Axmon et al. (2006) reported the outcome as fecundability (the likelihood of achieving pregnancy), we used the inverse odds ratio to describe the risk of infertility. This analysis revealed a significantly elevated summary OR of 1.15 (95 % CI 1.03–1.28) for infertility among HC. The subgroup analysis for questionnaire-based studies also produced a similar value (summary OR 1.18, 95 % CI 1.05–1.32). Begg’s test (*p* = 0.7194) and Egger’s test (*p* = 0.8893) did not reveal significant publication bias.

### Fetal death

**Fig. 5:**
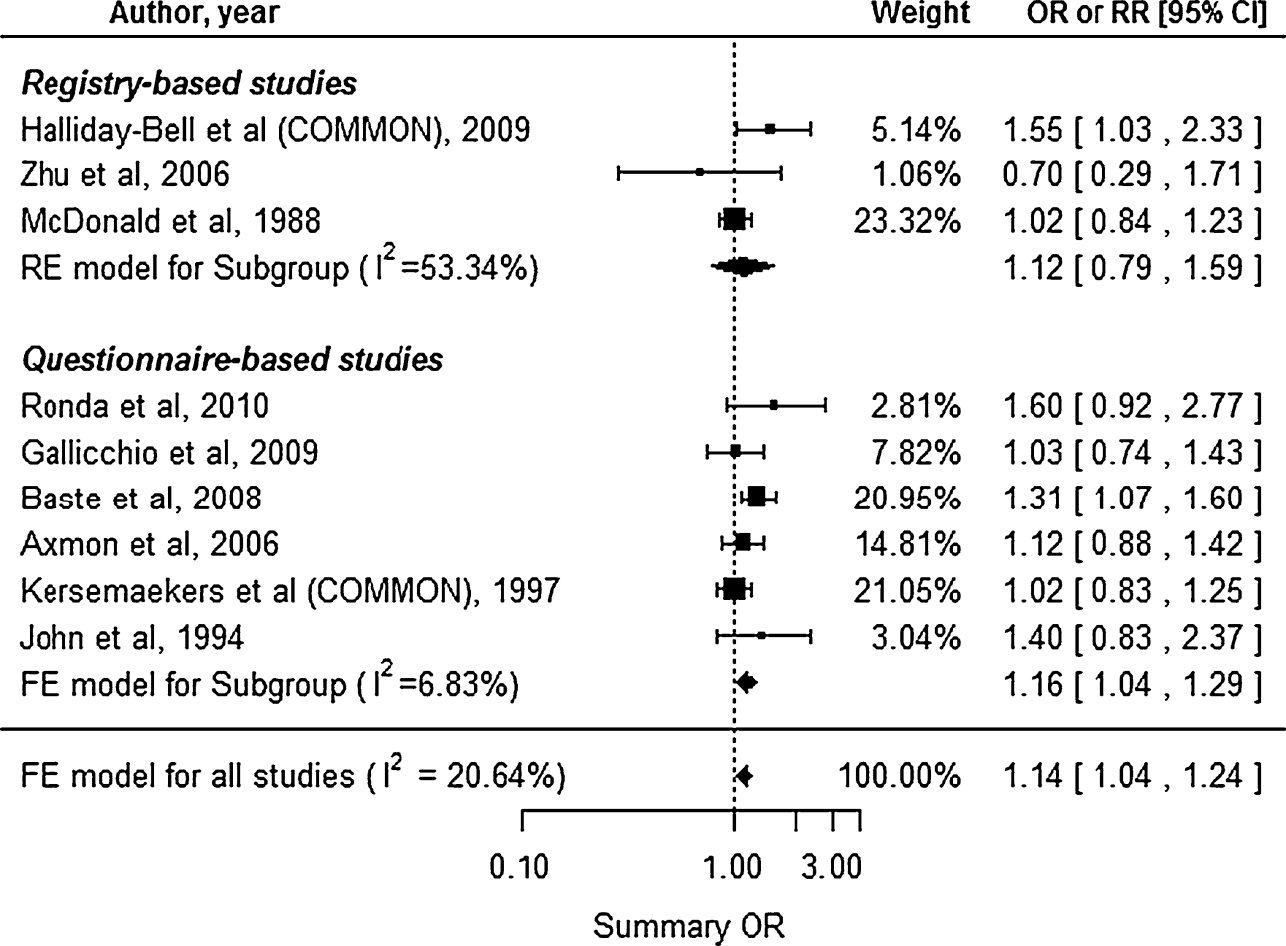
Forest plot for our meta-analysis of fetal death. *CI* confidence interval, *RE* random-effect, *FE* fixed-effect, *OR* odds ratio, *RR* relative risk, *COMMON* common risk estimated

Nine studies reported the fetal death outcome, including 3 registry-based and 6 questionnaire-based studies (Fig. 5). The studies by Halliday-Bell et al. (2009) and Kersemaekers et al. (1997) reported outcomes for two different groups or study periods, and we estimated the common risks for each of the studies. The studies were assumed to be homogenous, rather than heterogeneous (*I*^2^ = 20.64 %, Cochran’s *Qp* = 0.2594), and we used the FE model. The results revealed a significantly increased risk of fetal death among HC (summary OR 1.14, 95 % CI 1.04–1.24). The subgroup analysis for registry-based studies did not reveal a significantly increased risk (summary OR 1.12, 95 % CI 0.79–1.59), although the subgroup analysis for questionnaire-based studies did reveal a significantly increased risk (summary OR 1.16, 95 % CI 1.04–1.29). Begg’s test (*p* = 0.4767) and Egger’s test (*p* 0.4087) did not reveal significant publication bias.

### Preterm delivery

**Fig. 6:**
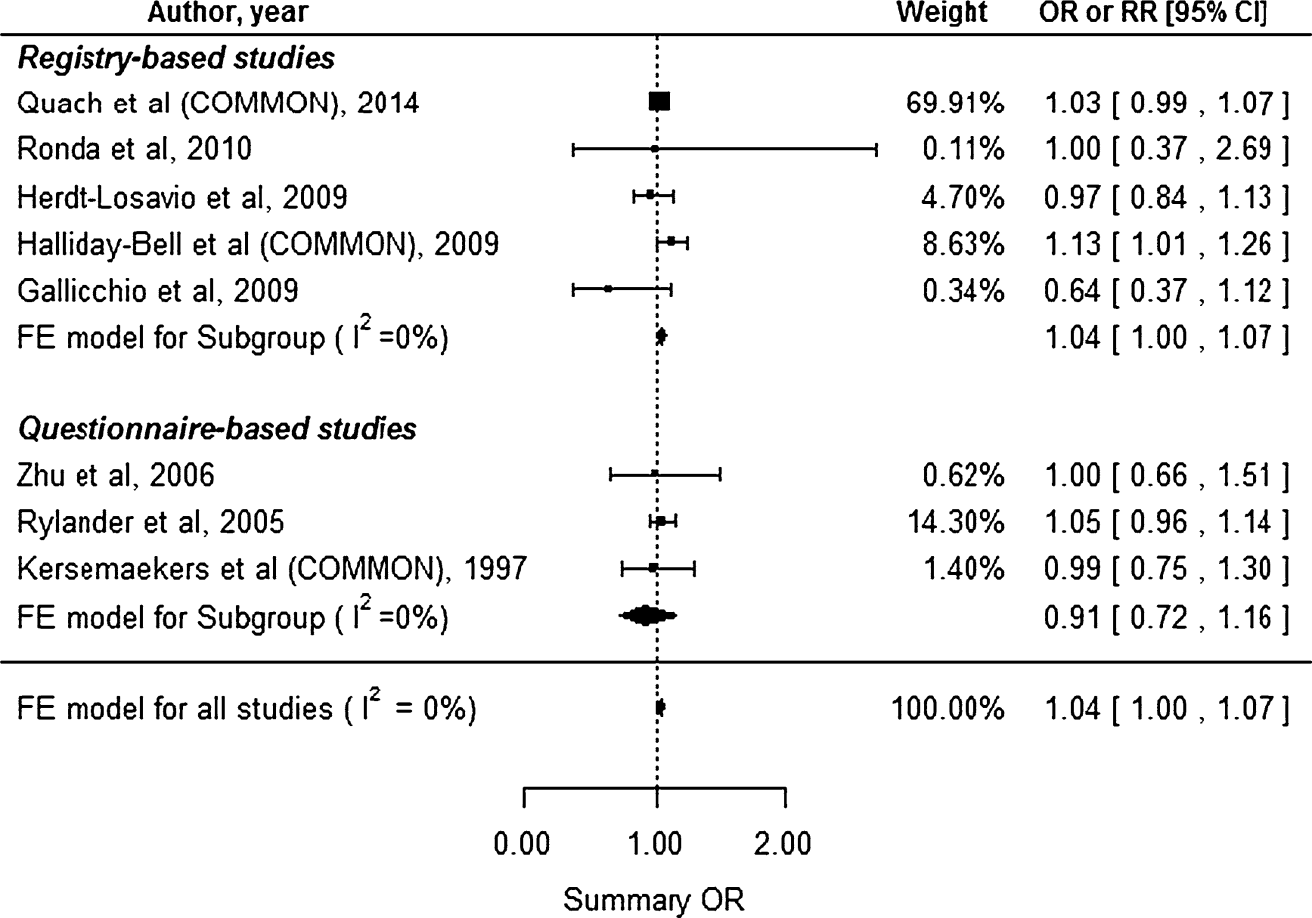
Forest plot for our meta-analysis of preterm delivery. *CI* confidence interval, *FE* fixed-effect, *OR* odds ratio, *RR* relative risk, *COMMON* common risk estimated

Eight studies reported the preterm delivery outcome, including 5 registry-based and 3 questionnaire-based studies (Fig. 6). The studies by Quach et al. (2014), Halliday-Bell et al. (2009), and Kersemaekers et al. (1997) described different exposure groups; therefore, we estimated the common risks for each of the studies. No substantial heterogeneity was observed (*I*^2^ = 0.0 %, Cochran’s *Qp* = 0.5065), and we used the FE model. This analysis revealed a significantly increased summary OR of 1.04 (95 % CI 1.00–1.07) for preterm delivery among HC. Subgroup analysis of the registry-based studies also revealed an significantly increased risk (summary OR 1.04, 95 % CI 1.00–1.07), although the subgroup analysis of questionnaire-based studies revealed an insignificantly decreased risk (summary OR 0.91, 95 % CI 0.72–1.16). Begg’s test (*p* = 0.9049) and Egger’s test (*p* = 0.4416) did not reveal significant publication bias.

### Subgroup analysis

Subgroup analyses were performed for occupation (cosmetologist/hairdresser), quality score with a cutoff of 7, the potential for recall bias, and study type (registry-/questionnaire-based). The manicurists in the study by Quach et al. (2014) were considered cosmetologists, while the study by Halliday-Bell et al. (2009) was evaluated as two separate studies in the occupation analysis. In the study type analysis, the risk for preterm delivery was significantly increased in registry-based studies, whereas those for infertility and fetal death were significantly increased in questionnaire-based studies. In the quality score analysis, the risk for preterm delivery was significantly increased in the high score group. In the occupation analysis (hairdresser/cosmetologist), all risks for the five reproductive outcomes were significantly increased in the hairdresser group, whereas those for the four reproductive outcomes were insignificantly increased in the cosmetologist group (Table 3).

**Table 3:** Summary of subgroup analysis according to study type, quality score, potential for recall bias, and study subjects

	SGA				LBW				Infertility				Fetal death				Preterm delivery			
	*N* ^a^	*I* ^2^ (%)	Model	Summary OR (95 % CI)	*N*	*I* ^2^ (%)	Model	Summary OR (95 % CI)	*N*	*I* ^2^ (%)	Model	Summary OR (95 % CI)	*N*	*I* ^2^ (%)	Model	Summary OR (95 % CI)	*N*	*I* ^2^ (%)	Model	Summary OR (95 % CI)
Total	7	87.15	RE	1.14 (0.97–1.33)	8	72.36	RE	1.12 (0.98–1.27)	6	42.71	FE	1.15* (1.03–1.28)	9	20.64	FE	1.14* (1.04–1.24)	8	0	FE	1.04* (1.00–1.07)
Study type																				
Registry-based	7	87.15	RE	1.14 (0.97–1.33)	5	81.03	RE	1.11 (0.97–1.27)	1	NA			3	53.34	RE	1.12 (0.79–1.59)	5	0	FE	1.04* (1.00–1.07)
Questionnaire-based	0	NA			3	45.52	FE	1.17 (0.88–1.56)	5	47.21	FE	1.18* (1.05–1.32)	6	6.83	FE	1.16* (1.04–1.29)	3	0	FE	0.91 (0.72–1.16)
Quality score																				
≥7	6	86.73	RE	1.12 (0.92–1.36)	6	78.66	RE	1.10 (0.89–1.35)	3	21.00	FE	1.17 (0.99–1.38)	7	13.06	FE	1.12 (0.99–1.27)	7	0	FE	1.03* (1.00–1.07)
<7	1	NA			2	NA			3	67.18	RE	1.06 (0.82–1.38)	2	NA			1	NA		
Potential for recall bias																				
Low	7	87.15	RE	1.14 (0.97–1.33)	6	77.55	RE	1.12 (0.98–1.29)	1	NA			4	50.51	RE	1.20 (0.89–1.64)	6	0	FE	1.04* (1.00–1.07)
High	0	NA			2	NA			5	47.21	FE	1.18* (1.05–1.32)	5	0	FE	1.14* (1.02–1.28)	2	NA		
Study subjects^b^																				
Hairdresser	5	69.47	RE	1.20* (1.00–1.45)	6	57.41	RE	1.20* (1.03–1.40)	5	20.48	FE	1.19* (1.06–1.32)	7	33.71	FE	1.13* (1.03–1.25)	5	0	FE	1.09* (1.02–1.16)
Cosmetologist	3	78.30	RE	1.11 (0.91–1.35)	3	41.94	FE	1.00 (0.96–1.05)	1	NA			3	0	FE	1.15 (0.88–1.49)	4	33.88	FE	1.02 (0.98–1.06)

*OR* odds ratio, *CI* confidence interval, *SGA* small for gestational age, *LBW* low birth weight, *RE* random-effect model, *FE* fixed-effect model, *NA* not available

* Statistical significance

^a^Number of included studies

^b^The study by Halliday-Bell et al. (2009) was evaluated as two separate studies

## Discussion

To our knowledge, this is the first meta-analysis of epidemiological studies to examine the risk of reproductive disorders among HC. A total of 19 eligible studies were included in our meta-analysis, and the results revealed that these workers had significantly increased risks of various reproductive disorders, including infertility, fetal death, and preterm delivery.

Previous studies have reported that cosmetology or hairdressing is associated with a variety of health issues, including malignancies in lung, larynx, and bladder (Takkouche et al. 2009); asthma (Moscato and Galdi 2006); chronic bronchitis and asthma-like symptoms (Leino et al. 1997; Brisman et al. 2003); and contact dermatitis (Uter et al. 1999; Lee and Nixon 2001; Khumalo et al. 2006; Lind et al. 2007). Nevertheless, no studies have conclusively reported reproductive risks among HC, although SGA, LBW, and spontaneous abortions have been frequently investigated. In addition, other studies have described increased risks of infertility, congenital malformations, SGA, LBW, and cancer during childhood. Furthermore, one systematic review has reported that an increased risk of fertility disorders and pregnancy complications among HC cannot be excluded (Peters et al. 2010), although the risk of reproductive disorders was thought to be low. Similarly, our analysis revealed significantly increased risks of 15 % for infertility, 14 % for fetal death, and 4 % for preterm delivery when we compared HC to other populations or occupational groups.

The studies that we evaluated used different methodological approaches, which make it difficult to draw a definitive conclusion regarding our findings. For example, it is possible that patients were misclassified in the original studies due to the reliance on recall and that recall bias may have affected our findings, because mothers of children with adverse outcomes may have better recall of exposures, due to their heightened awareness. However, variables such as smoking, drinking, and drug use may be underreported because of the stigma that is associated with these behaviors, especially when the subject is pregnant (Reichman and Hade 2001). Although the time to pregnancy was likely accurately reported by the women (Peretz et al. 2009), the recall regarding work-related factors in the hairdresser cohort may not have been equally good. Nevertheless, if misclassification was introduced via the studies’ questionnaires, it may have caused underestimation of the effects of specific exposures. To avoid misclassification of exposure, detailed questions were asked about specific tasks (Herdt-Losavio et al. 2011), and birth certificates were used to validate information that was given by the participants regarding birth weight and certain potential confounders (Rylander and Källén 2005; Zhu et al. 2006; Halliday-Bell et al. 2009; Herdt-Losavio et al. 2009; Li et al. 2010). In our analysis, it is possible that methodological differences affected the results of the analyses, although we performed subgroup analyses for the registry- and questionnaire-based studies, which revealed few significant differences. However, one notable exception was the decreased risk of preterm delivery in the questionnaire-based studies compared to the registry-based studies, although it was not statistically significant.

Another potential source of bias is the use of different reference groups, as the ideal reference group would include women with similar background and working conditions (compared to HC), which would minimize any potential confounding via socioeconomic factors or personal cosmetics use. However, several studies used a single occupational group as the reference group, which included teachers, realtors, shop assistants, and office workers (Halliday-Bell et al. 2009; Herdt-Losavio et al. 2009; Ronda et al. 2010). In contrast, other studies used various occupational groups to provide a more robust comparison to HC (McDonald et al. 1987, 1988; Axmon and Rylander 2009; Gallicchio et al. 2009; Peretz et al. 2009; Li et al. 2010; Quach et al. 2014). The Swedish studies used all newborns or a sample from the general population (Rylander and Källén 2005; Li et al. 2010), the Dutch study used clothing sales clerks (Kersemaekers et al. 1997), and the Danish study used shop assistants and receptionists (Zhu et al. 2006).

Moreover, job title was used as a proxy for exposure in some studies, although the hairdressing or cosmetology occupations may not be synonymous with exposure to adverse chemicals or work conditions. Exposure assessment is a critical aspect of occupational studies, although the exact assessment techniques can vary for individual studies and range from a simple designation of “cosmetologist” or “hairdresser” as the occupation (McDonald et al. 1987, 1988; Rylander and Källén 2005; Li et al. 2010; Quach et al. 2014) to using questionnaire information for exposure assessment according to task and working hours (e.g., practice vs. shop assistant and office work, and full time vs. part time work) (Hougaard et al. 2006; Zhu et al. 2006; Herdt-Losavio et al. 2011; Ronda et al. 2009, 2010; John et al. 1994), and to differentiation according to the individual hair cosmetic products that are used (Peters et al. 2010). In this context, occupation alone only provides a rough estimation of exposure, and the subsequent risk assessment is likely inaccurate.

Another essential aspect of exposure assessment is the study period, as hairdressers’ exposure to chemicals varies widely over different time periods, which complicates measurement and analysis for occupational groups such as hairdressing. However, legislated regulations have led to changes in beauty shops’ working environments, such as their sources of exposure and protective facilities. Kersemaekers et al. (1997) assessed the time periods before and after regulatory changes in the Netherlands and reported that the risks of pregnancy complications decreased over time. The authors attributed this decreased risk to the exchange of toxic agents in beauty salons for less hazardous alternatives.

HC work in a complex environment with several factors that might affect female reproductive function, although chemical exposure has been most frequently mentioned as the cause of reproductive risk among HC in most studies (Ronda et al. 2010). More than 9000 chemicals are found in cosmetic products (Halliday-Bell et al. 2009), including nitrosamines in hair dye, toluene in nail polish, and phthalates in both hair dye and nail polish (Pak et al. 2013). Exposure to volatile organic compounds (VOCs) such as toluene (inhaled from paint reducer or paint thinner) during pregnancy has adverse effects on the neonate, including intrauterine growth retardation, premature delivery, congenital malformations, and postnatal developmental retardation (Donald et al. 1991). In addition, Peretz et al. (2009) have suggested that environmental exposure to chemicals, such as selenium, ethylene glycol monomethyl ether, and phenylenediamine, may be associated with poor reproductive function and reduced fertility. These chemicals are either inhaled as volatile compounds or absorbed by the skin, as HC often handle them manually. The exposure can accumulate if the products are used daily or if poor ventilation exists in salons (Mendes et al. 2011). Calculations of baseline values for exposure among French hairdressers working in small hairdressing salons revealed that dermal and inhalation exposure can reach 14.68 and 18.1 mg/kg/day, respectively (Ramirez-Martinez et al. 2015). In a study that was conducted in Italy, products used in hair salons generated an average airborne formaldehyde concentration of 2.4 ppm during heat treatment of hair at 230 °C, and the 8-h exposure level of hairdressers ranged from 0.1 to 0.4 ppm, depending on the number of daily treatments (1–4) (Grana et al. 2013). Another study that investigated the chemical exposure level among Portuguese hairdressers revealed that the average concentration of total VOCs was 1.4 mg/m^3^ above the Portuguese reference level (0.6 mg/m^3^) and that 4 % of hairdressers had a mean NH_3_ concentration that was higher than the Portuguese (20 ppm) and American Conference of Governmental Industrial Hygienists (ACGIH) (25 ppm) reference levels (Mendes et al. 2011).

Long working hours and standing throughout the working period have also been associated with higher incidences of spontaneous abortion, SGA, LBW, and preterm birth (Mozurkewich et al. 2000; Bonzini et al. 2007). Palmer et al.’s meta-analysis revealed that an increased risk of preterm delivery was associated with working >40 h per week (a 23 % increase) and standing at work for 4 h per day (a 22 % increase) (Palmer et al. 2013). Exposure to stressful occupational conditions may interfere with a woman’s endocrine system, which may explain these adverse reproductive effects (Dole 2003). Another potential explanation for the association between stress and reproductive outcomes (specifically fetal death) is that women who experience high levels of stress are more likely to be smokers (Nelson et al. 2003).

## Conclusion

The results of our meta-analysis suggest that there is a significant increase in the risk of reproductive disorders among HC, compared to the general population or other occupational groups. However, these results should be interpreted within the context of the potential for bias in our findings. Therefore, further studies are needed to evaluate the specific risk factors that are associated with the hairdressing and cosmetology occupations and their adverse effects on reproductive health. In this context, the risk for HC is low when considered from an absolute perspective, although HC are common throughout the world and many of them are women who are of reproductive age. Therefore, the 4–15 % risk increase among HC may be important from the public health perspective. These concepts suggest that improvements in occupational health and safety could reduce the considerable incidence of reproductive disorders in this population. To achieve this goal, we believe that multi-disciplinary efforts should involve health and safety professionals, epidemiologists, engineers, social scientists, and ergonomists, in order to make HC a safer occupation.
